# Digital Engagement in Diabetes Care: A Multi-Domain Analysis of Psychosocial and Clinical Determinants

**DOI:** 10.3390/healthcare14060800

**Published:** 2026-03-21

**Authors:** Mirela Frandes, Adriana Gherbon, Bogdan Timar, Cǎlin Muntean

**Affiliations:** 1 Department of Functional Sciences—Medical Informatics and Biostatistics, “Victor Babes” University of Medicine and Pharmacy, 300041 Timisoara, Romania; mirela.frandes@umft.ro (M.F.); cmuntean@umft.ro (C.M.); 2 Centre for Modeling Biological Systems and Data Analysis, “Victor Babes” University of Medicine and Pharmacy, 300041 Timisoara, Romania; 3 Department VII Internal Medicine—Diabetes, Nutrition, Metabolic Diseases and Systemic Rheumatology, “Victor Babes” University of Medicine and Pharmacy, 300041 Timisoara, Romania; bogdan.timar@umft.ro; 4 Centre of Molecular Research in Nephrology and Vascular Disease, “Victor Babes” University of Medicine and Pharmacy, 300041 Timisoara, Romania; 5 Diabetes, Nutrition, and Metabolic Diseases, “Pius Brinzeu” Emergency Hospital, 300723 Timisoara, Romania

**Keywords:** diabetes, digital engagement, beta regression, psychosocial determinants, self-efficacy, technology acceptance model, stratified analysis

## Abstract

**Background:** The growing use of digital health technologies in diabetes care offers new opportunities for self-management and clinical monitoring. However, there remains significant variability in the extent to which individuals engage with these digital tools. Understanding the psychosocial and clinical factors associated with the use of digital health technologies is crucial for developing targeted implementation strategies. **Objectives:** The aim of this study was to assess the use of digital health technologies among adults with diabetes and to explore their relationship with psychosocial factors—especially technology acceptance and self-efficacy—as well as certain clinical characteristics, including diabetes-related stress, age, and disease duration. **Methods:** We conducted a cross-sectional study involving 304 adults with diabetes. Digital engagement was measured using the Digital Adherence and Use Questionnaire (DAUQ), a 7-item self-report instrument (Cronbach’s α = 0.89), from which a composite Digital Engagement Score was calculated (range 1–5) to indicate the level of technology-related self-management behaviors. Participants were descriptively categorized into low- and high-engagement groups. Engagement patterns were also analyzed by diabetes type to understand structural differences in technology exposure. Relationships between psychosocial variables and the outcome were examined using correlation analyses. Since engagement among participants with type 1 diabetes (T1D) showed limited variability, multivariable regression analyses were performed on participants with type 2 diabetes (T2D) using beta regression, with linear regression as a sensitivity analysis. An exploratory beta regression was also conducted for T1D. **Results:** Overall, 35.5% of participants were classified as having high digital engagement. High engagement was observed in more than 90% of participants with T1D, compared to 4.1% of those with T2D. Median engagement scores differed significantly between low- and high-engagement groups (median [Q1–Q3]: 1.71 [1.71–2.39] vs. 3.86 [3.86–4.43]). Highly engaged participants reported much higher levels of openness to technology (median [Q1–Q3]: 5.00 [1.00–5.00] vs. 1.00 [1.00–1.00], *p* < 0.001) and self-efficacy (median [Q1–Q3]: 3.00 [3.00–3.00] vs. 5.00 [5.00–5.00], *p* < 0.001). In T1D, multivariable beta regression analyses showed that age was independently associated with digital engagement, with each 10-year increase corresponding to a decrease in engagement (β = −0.147, 95% CI −0.219 to −0.075, *p* < 0.001). Diabetes duration and psychosocial variables were not independently associated with engagement in the multivariable model. In contrast, among participants with T2D, insulin treatment emerged as the strongest independent predictor of engagement (β = 0.996, 95% CI 0.859–1.134, *p* < 0.001), and diabetes-related stress emerged as an independent predictor of engagement (β = 0.069, 95% CI 0.006–0.132, *p* = 0.033). Technology acceptance was positively associated with engagement (β = 0.694, 95% CI 0.350–1.037, *p* < 0.001), whereas higher self-efficacy was independently associated with lower engagement intensity (β = −0.366, 95% CI −0.608 to −0.124, *p* = 0.003). Age and diabetes duration were not independently associated with engagement after adjustment. **Conclusions:** Digital engagement appears to function as a structurally embedded component of self-management in T1D, with limited variability and largely independent of psychosocial modulation. In T2D, engagement is predominantly driven by treatment characteristics (insulin treatment), psychosocial dynamics (stress, technology acceptance), with higher self-efficacy associated with reduced reliance on digital tools. These findings suggest distinct behavioral mechanisms underlying digital health utilization across diabetes types and support the need for tailored implementation strategies.

## 1. Introduction

The integration of digital health technologies into diabetes care has expanded rapidly over the past decade, offering new tools for glucose monitoring, self-management support, and communication between patients and healthcare providers. Mobile health applications, continuous glucose monitoring systems, and telemedicine platforms have been associated with improvements in glycemic outcomes and self-care behaviors in both type 1 diabetes (T1D) and type 2 diabetes (T2D) [1,2,3,4]. Consequently, digital health solutions are increasingly promoted as essential components of contemporary diabetes management strategies [5].

Despite increasing availability, the actual use of digital health technologies varies widely in real-world settings. While some individuals rely heavily on digital tools for daily disease management, others exhibit limited or inconsistent engagement, even when access barriers are few [6,7,8,9]. This variation underscores the gap between simply having technology and using it in a meaningful way. It indicates that factors beyond clinical need alone may affect engagement patterns.

These challenges are not unique to diabetes. Analogous patterns of variable digital engagement have been documented across multiple chronic conditions. In cardiovascular disease, mobile health interventions have shown significant heterogeneity in adherence and clinical outcomes [10,11]. In chronic respiratory disease, digital self-management tools demonstrated differential uptake across patient subgroups [12]. In oncology, patient engagement with electronic symptom monitoring varied substantially by demographic and psychosocial characteristics [13]. These cross-disease findings suggest that the determinants of digital health engagement are not disease-specific but reflect broader behavioral and structural dynamics that merit integrated investigation.

Psychosocial factors are increasingly seen as key drivers of digital health behaviors. The Technology Acceptance Model (TAM) emphasizes perceived usefulness and perceived ease of use as critical predictors of technology-related actions. It has been extensively applied in healthcare settings [14,15,16]. In managing chronic diseases, where continuous interaction with digital tools is required, these attitudinal factors can be especially influential.

Self-efficacy represents another important construct in understanding engagement with digital health technologies. Higher levels of self-efficacy have been consistently associated with improved diabetes self-management behaviors, including treatment adherence, glucose monitoring, and lifestyle modification [17,18,19]. Individuals who feel confident in their ability to manage diabetes may be more inclined to adopt and maintain the use of digital tools that support self-care. In contrast, lower self-efficacy may hinder engagement despite perceived benefits.

Clinical characteristics such as age and diabetes duration have also been examined as potential determinants of digital health use. Although younger age is often assumed to predict greater technology use, findings across studies have been inconsistent [15,16,20]. Similarly, longer disease duration does not necessarily translate into greater engagement with digital tools, suggesting that experience with diabetes management alone may be insufficient to account for observed usage patterns [21]. Emotional burden, including diabetes-related stress and distress, adds further complexity, as it may either motivate increased engagement or act as a barrier to sustained use [22,23,24,25].

Importantly, much of the existing literature has relied on binary indicators of technology use or has examined psychosocial and clinical determinants in isolation, limiting the ability to capture meaningful variability in engagement intensity [25,26,27]. There remains a need for integrated analytical approaches that conceptualize digital engagement as a behavioral continuum and simultaneously account for psychosocial and clinical factors.

Despite the growing body of literature on digital health use in diabetes, several methodological gaps persist. Most studies have employed binary indicators of technology adoption (use vs. non-use), limiting the ability to capture meaningful gradations in engagement intensity. Few have examined psychosocial and clinical determinants simultaneously within an integrated analytical framework, and stratified analyses that account for the fundamentally different care structures of T1D and T2D remain scarce. Moreover, the application of distributional models appropriate for bounded, continuous engagement outcomes—such as beta regression—has been largely absent from this literature. The present study addresses these gaps by modeling digital engagement as a continuous behavioral outcome using beta regression, by simultaneously incorporating technology acceptance, self-efficacy, and diabetes-related stress alongside clinical variables, and by stratifying all analyses by diabetes type to reveal potentially distinct determinants of engagement.

Therefore, the present study aimed to evaluate the use of digital health technologies among adults with diabetes and to examine their association with psychosocial factors, including technology acceptance and self-efficacy, as well as selected clinical characteristics, such as diabetes-related stress, age, and disease duration. By adopting an integrated analytical approach, this study seeks to provide a more comprehensive understanding of the determinants of real-world digital engagement in diabetes care.

## 2. Materials and Methods

### 2.1. Study Design and Participants

A cross-sectional observational study was conducted between January and June 2025 in three outpatient diabetes clinics in Western Romania. The target population comprised adults (≥18 years) with physician-confirmed T1D or T2D receiving routine outpatient care. Exclusion criteria included severe cognitive impairment (Mini-Mental State Examination score < 24), terminal illness, or inability to provide informed consent. The final analytic sample included 304 participants (T1D: *n* = 104; T2D: *n* = 200). Ethical approval was obtained from the Victor Babes University Ethics Committee (Protocol No. 47/2025), and all participants provided written informed consent before study participation.

### 2.2. Digital Engagement (Digital Adherence and Use Questionnaire—DAUQ)

Digital engagement was measured using the Digital Adherence and Use Questionnaire (DAUQ), a structured self-report tool designed to capture patients’ real-world use of digital health technologies. The DAUQ emphasizes behavioral engagement by evaluating how digital tools are integrated into routine self-management practices, rather than focusing on attitudes, intentions, or perceived usefulness. The questionnaire includes several items addressing key areas of digital health use in diabetes care, including glucose-monitoring devices, mobile health apps, and digital platforms for disease tracking and communication. Participants rated how often and how consistently they engaged with these technologies using Likert-scale response options.

A composite Digital Engagement Score was derived by the arithmetic mean of 7 items, yielding a continuous score ranging from 1 to 5 (1 = never, 5 = very often), with higher values indicating greater intensity of digital engagement. The observed range was 1.71–4.86, and no items were skipped (100% completion rate across all 304 participants); therefore, no missing-data imputation was required. The seven DAUQ core functions were:

1. Glucose value logging
2. Meal/carbohydrate tracking
3. Insulin/medication dose tracking
4. Physical activity monitoring
5. Treatment reminder use
6. Trend visualization
7. Data sharing with healthcare providers

This score was treated as a continuous variable to reflect engagement as a behavioral spectrum rather than a binary measure. The DAUQ demonstrated strong internal consistency (Cronbach’s α = 0.89). Importantly, the continuous engagement score was retained for analysis rather than being split at the median, thereby preserving statistical power and avoiding the artificial categorization of a naturally continuous construct [14]. For descriptive purposes, participants were also divided into low- and high-engagement groups based on the distribution of the Digital Engagement Score. This classification was used only for descriptive and comparative analyses and did not replace the continuous engagement measure used in regression modeling.

### 2.3. Psychosocial Measures

Psychosocial factors influencing digital engagement were measured using established self-report instruments assessing technology attitudes, perceived skills, and diabetes-related stress. These factors were selected based on prior research linking psychosocial factors to technology use and self-management among populations with chronic illnesses.

Technology acceptance was assessed using a 9-item Romanian adaptation of the Technology Acceptance Model (TAM), focusing on perceived usefulness and perceived ease of use of digital health technologies. The Romanian-adapted TAM scale assessed perceived usefulness, perceived ease of use, trust in data security, behavioral intention, openness to new technologies, and preference for digital control. Responses were collected using a 5-point Likert scale (1 = strongly disagree, 5 = strongly agree), with higher scores indicating greater acceptance of technology. Internal consistency was excellent (Cronbach’s α = 0.89). For regression analyses, a composite TAM score was computed as the mean of all items.

Self-efficacy was measured using a single-item self-rated competence scale (“How confident are you in your ability to manage your diabetes on a daily basis?”), rated on a 5-point Likert scale (1 = not at all confident to 5 = very confident). Single-item self-efficacy measures have demonstrated adequate validity in chronic disease populations when assessing global perceived competence [28]. Diabetes-related stress was measured using a single-item numeric rating scale (“On a scale of 0 to 10, how much stress does your diabetes cause you?”; 0 = no stress, 10 = extreme stress). Single-item stress measures have been used in diabetes distress screening contexts and correlate well with multi-item instruments [29]. These psychosocial measures were treated as independent explanatory variables and were conceptually and analytically distinct from the DAUQ-derived Digital Engagement Score, which captured observed patterns of technology use rather than attitudes or perceptions.

### 2.4. Clinical Variables

Clinical and treatment-related variables were collected to provide context for digital engagement patterns and to describe the clinical background of the study population. These variables were not meant to be main predictors of digital engagement but were included to account for differences in disease characteristics and care pathways that could influence technology exposure and use. Collected clinical variables included diabetes type, duration of diabetes (years since diagnosis), and current treatment modality, such as insulin therapy, continuous glucose monitoring (CGM), and insulin pump use, when applicable. These variables were used for descriptive purposes and in stratified analyses to explore structural differences in digital engagement, particularly with respect to diabetes type and treatment intensity.

### 2.5. Statistical Analysis

Statistical analyses were performed in accordance with a predefined analytical plan aligned with the study objectives. Continuous variables were summarized using medians and interquartile ranges (Q1–Q3), whereas categorical variables were presented as frequencies and percentages. Between-group comparisons of continuous variables used the Mann–Whitney U test (or Kruskal–Wallis test, as appropriate), due to the non-normal distribution of most measures. Comparisons of categorical variables employed the chi-square test or Fisher’s exact test, as appropriate. Initial descriptive analyses compared participant characteristics across digital engagement levels (low vs. high). To better understand engagement patterns, additional descriptive analyses were performed by diabetes type. Relationships between digital engagement and psychosocial variables were analyzed with Spearman’s rank correlation coefficients.

Psychosocial variables, including technology acceptance and self-efficacy, were included as explanatory variables in multivariable models, whereas diabetes-related stress was included as a covariate given its potential influence on self-management behaviors. Clinical variables were considered for contextual adjustment where appropriate. Model estimates were reported as regression coefficients with corresponding 95% confidence intervals.

Given the bounded nature of the DAUQ engagement score (theoretical range: 1–5), beta regression was selected as the primary analytic approach. Beta regression appropriately models continuous outcomes constrained to a finite interval (a, b). We applied the transformation:

$$y^{*}=\frac{y-a}{b-a}$$

where y represents the original DAUQ score, a = 1, and b = 5. The transformed variable y* follows a beta distribution on (0,1) with probability density function:

$$f(y^{*}|\mu ,\phi )=\frac{\Gamma (\phi )}{\Gamma (\mu \phi )\Gamma ((1-\mu )\phi )}(y^{*}{)}^{\mu \phi -1}(1-y^{*}{)}^{(1-\mu )\phi -1}$$

where μ ∈ (0,1) represents the mean parameter and φ > 0 the precision parameter. The link function (logit) relates predictors to the mean

$$g(\mu_{i})=log\left(\frac{\mu_{i}}{1-\mu_{i}}\right)=\beta_{0}+\beta_{1}X_{1i}+\cdots +\beta_{k}X_{ki}$$

Boundary value assessment: No DAUQ composite scores exactly matched the boundaries of 1.00 or 5.00 (observed range: 1.71–4.86). As a conservative measure, the Smithson–Verkuilen transformation was applied [30]. It is important to note that the DAUQ composite, which is an arithmetic mean of 7 Likert items, can only produce 29 theoretically possible values, with 8 observed in the data. This inherent discreteness presents a distributional limitation that standard beta regression does not explicitly address; the implications are discussed in the Limitations section.

Initial exploration revealed a nearly complete separation between diabetes types and engagement levels (T1D: 96.3% with DAUQ > 3.5; T2D: 98.5% with DAUQ < 3.0), causing significant confounding in combined analyses. Therefore, all regression models were stratified by diabetes type (T1D vs. T2D). 

Analytic plan:

1. Descriptive analyses: Distribution of continuous DAUQ engagement score visualized via kernel density plots stratified by diabetes type. Baseline characteristics were compared between T1D and T2D groups using the Mann–Whitney U test (continuous, non-Gaussian) and χ^2^ tests (categorical).
2. Correlation analyses: Spearman rank correlations between DAUQ engagement score and continuous variables, computed separately within each diabetes type group.
3. Primary regression analyses: Beta regression models were fitted separately for the T1D and T2D strata, with DAUQ engagement score as the outcome. Covariates included:
  ○ Demographic: age, sex, education level;
  ○ Clinical: diabetes duration, insulin treatment;
  ○ Psychosocial: self-efficacy, TAM composite score, diabetes-related stress.

Beta regression was used to model the bounded engagement score appropriately.

4. Sensitivity analyses:
  ○ Linear regression with untransformed DAUQ score to assess robustness to distributional assumptions;
  ○ Quantile regression (τ = 0.25, 0.50, 0.75) to examine predictor effects across the engagement distribution.

All statistical analyses were performed using R statistical software (version 4.3.2; R Core Team, Vienna, Austria), and a two-sided *p*-value < 0.05 was considered statistically significant. Model diagnostics involved assessing residuals using the DHARMa (Diagnostics for HierArchical Regression Models Alternative) package and calculating Variance Inflation Factors (VIFs) to assess multicollinearity (threshold: VIF > 5). All regression coefficients are presented with 95% confidence intervals and *p*-values. Beta regression coefficient effect sizes were first interpreted on the logit scale and subsequently converted back to the DAUQ metric to aid clinical interpretation.

## 3. Results

### 3.1. Distribution of Digital Engagement and Baseline Characteristics

Baseline demographic, clinical, digital health, and psychosocial characteristics of the study population, stratified by level of digital engagement, are shown in Table 1. Digital engagement was defined as the frequency of use of diabetes-related digital technologies, and participants were classified into low (*n* = 196; 64.5%) and high (*n* = 108; 35.5%) digital engagement groups based on the median DAUQ-derived engagement score (median = 2.71).

Participants with lower digital engagement were significantly older (67 vs. 40 years; *p* < 0.001). They also had lower educational levels compared to those with higher engagement (Table 1). Only 15.8% of the low-engagement group had higher education (university level), versus 44.4% of the high-engagement group. Clinical characteristics differed between groups: T2D was more common in participants with low engagement (100.0% vs. 3.7%), but both types of diabetes appeared across engagement categories in the overall population. The use of digital health tools, including telemedicine services (67.9% vs. 95.4%), CGM, and insulin pumps, was observed in both groups, indicating that low digital engagement reflected reduced use rather than complete non-use of digital technologies.

Pronounced differences were observed in psychosocial variables. Participants with low digital engagement reported significantly lower self-efficacy (3.00 vs. 5.00, *p* < 0.001), perceived usefulness (1.00 vs. 5.00), and perceived ease of use of digital technologies (1.00 vs. 5.00), as well as lower diabetes-related stress scores (3.00 vs. 5.00, *p* < 0.001) compared to those with higher engagement (Table 1). The classification of low versus high engagement largely reflects underlying clinical pathways rather than voluntary adoption alone.

### 3.2. Digital Engagement Patterns by Diabetes Type

**Figure 1:**
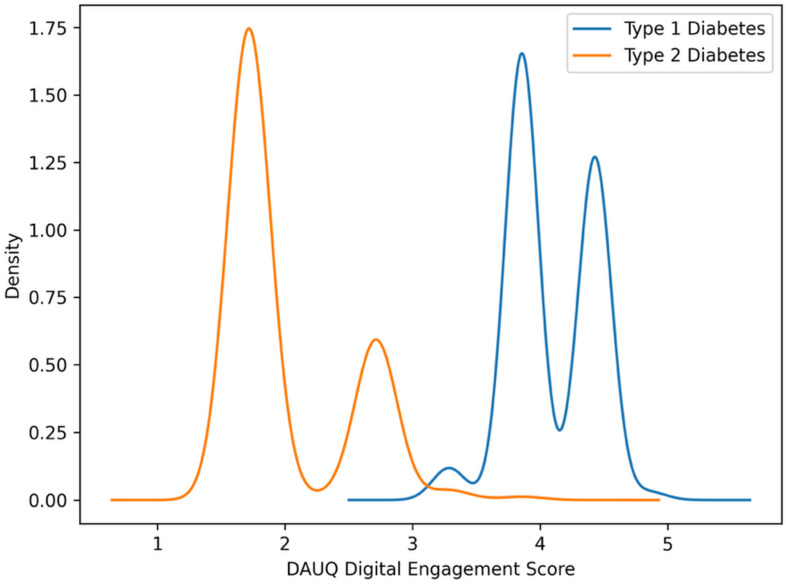
Distribution of the DAUQ-based Digital Engagement score in the study population.

Given the significant imbalance in digital engagement between the low- and high-engagement groups, we further analyzed engagement patterns based on diabetes type to understand the underlying clinical and care-related structures. We found that the DAUQ-derived engagement score showed a bimodal distribution, which was strongly linked to diabetes type (Figure 1).

T1D participants showed significantly higher median engagement intensity (3.86, 3.86–4.43) compared to T2D participants (1.71, 1.71–2.71), *p* < 0.001. T1D participants were significantly younger (38.5 vs. 67.5 years; *p* < 0.001), had higher educational levels (university: 44.4% vs. 15.8%; *p* < 0.001), and scored significantly higher on psychosocial measures: self-efficacy (5.0, 5.0–5.0 vs. 3.0, 3.0–3.0; *p* < 0.001), technology acceptance composite (5.0, 5.0–5.0 vs. 3.0, 3.0–3.0; *p* < 0.001), and diabetes-related stress (6.0, 6.0–6.0 vs. 3.0, 3.0–3.0; *p* < 0.001). CGM use was nearly universal among T1D participants (93.5%) but absent among T2D participants (0.0%), reflecting Romanian reimbursement policies that mainly restrict CGM access to T1D patients (Table 2).

These observed differences reflect structural features of diabetes care, with technology-intensive treatment routes in T1D leading to consistently high engagement and minimal variability.

### 3.3. Associations Between Digital Engagement and Psychological Factors

Spearman correlations between the DAUQ engagement score and study variables, calculated separately for each type of diabetes, are presented in Table 3. In the T1D group, engagement intensity had moderate positive correlations with self-efficacy (r = 0.379, *p* < 0.001) and the technology acceptance score (r = 0.379, *p* < 0.001), while the correlation with diabetes-related stress was weaker (r = 0.187, *p* = 0.058). Age was moderately negatively correlated (r = −0.452, *p* < 0.001). In the T2D group, the correlations were much weaker: self-efficacy (r = −0.032, *p* = 0.654) and the TAM composite (r = 0.075, *p* = 0.291), although diabetes-related stress showed a strong correlation (r = 0.866, *p* < 0.001).

**Figure 2:**
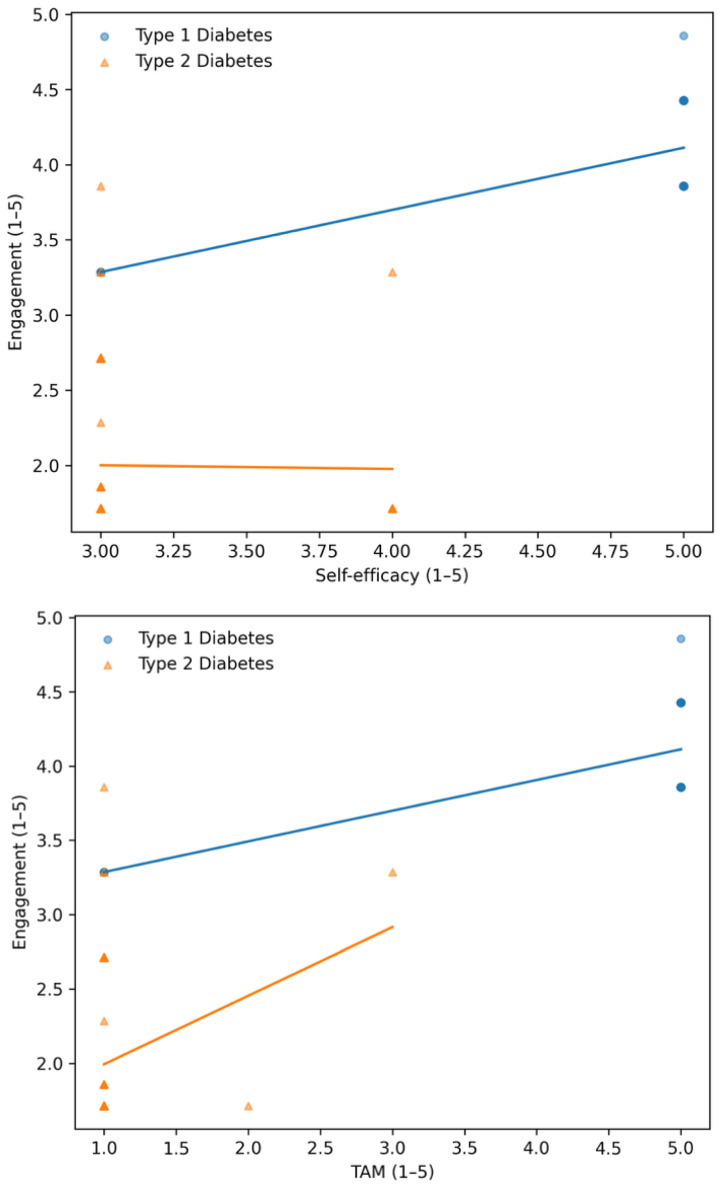

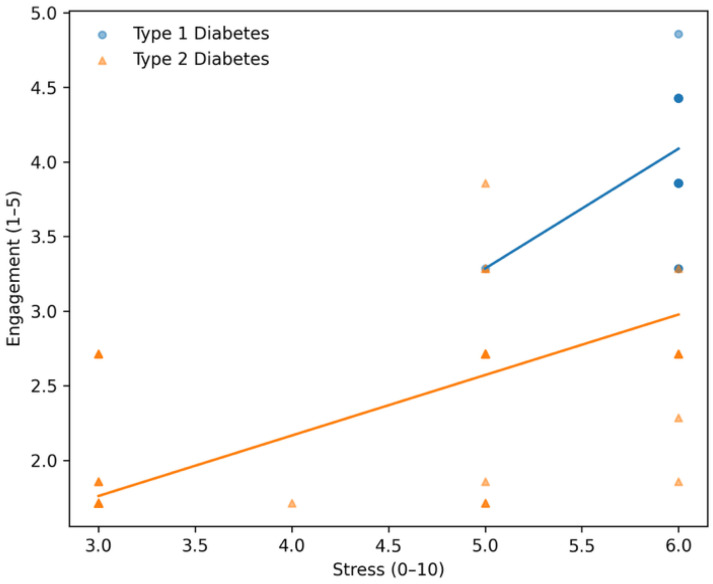
Association between digital engagement and psychosocial characteristics.

Figure 2 illustrates the connection between digital engagement and technology acceptance. Higher levels of digital engagement correlate with higher TAM scores, indicating that perceived usefulness, ease of use, and willingness to continue using digital technologies are positively associated with actual usage patterns.

### 3.4. Multivariable Analysis of Digital Engagement Intensity

The beta regression model for T2D is presented in Table 4. Insulin treatment was the strongest predictor of engagement intensity (β = 0.996, 95% CI: 0.859–1.134, *p* < 0.001), suggesting that T2D participants on insulin therapy had significantly higher digital engagement. Technology acceptance was independently and positively related to engagement (β = 0.694, 95% CI: 0.350–1.037, *p* < 0.001). Self-efficacy was inversely related to engagement (β = −0.366, 95% CI: −0.608 to −0.124, *p* = 0.003). Diabetes-related stress remained positively associated with engagement, although the impact was greatly reduced after adjusting for insulin treatment (β = 0.069, 95% CI: 0.006–0.132, *p* = 0.033). Demographic factors such as age, sex, education, and diabetes duration were not independently linked to engagement in the full model.

To illustrate the clinical significance of these effects on the original DAUQ scale, predicted mean engagement scores were calculated at typical covariate values. In the full model, T2D participants on insulin had a predicted mean DAUQ of about 2.4, compared to 1.7 for those not on insulin, with other covariates held at median values. A one-unit increase in self-efficacy (from 3 to 4) was linked to an estimated decrease of roughly 0.25 points on the DAUQ scale.

**Figure 3:**
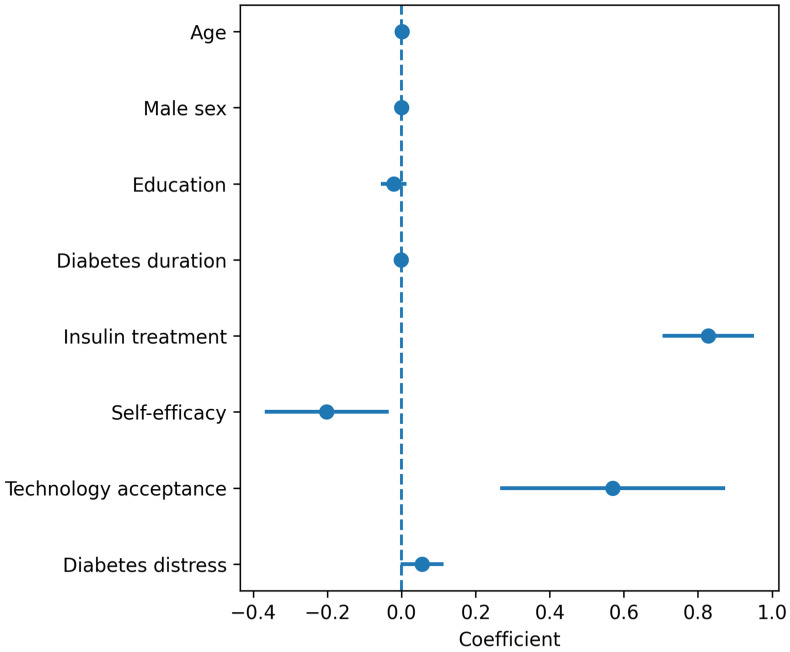
Psychosocial and clinical predictors of digital engagement among participants with type 2 diabetes.

In a reduced sensitivity model excluding sex, education, and insulin treatment, diabetes-related stress was the strongest predictor (β = 0.479, 95% CI: 0.436–0.522, *p* < 0.001), with TAM (β = 0.603, *p* = 0.013) and self-efficacy (β = −0.588, *p* < 0.001) also significant. The substantial change in the stress coefficient between models (from 0.479 to 0.069) indicates that much of the stress–engagement association was confounded by insulin treatment status. A forest plot displaying beta regression coefficients and 95% confidence intervals is shown in Figure 3. All major predictors included in the multivariable beta regression model are presented, with the DAUQ-derived digital engagement score as a continuous outcome. Technology acceptance and self-efficacy demonstrated larger effect sizes, while diabetes-related stress, diabetes duration, and age showed smaller effect sizes. 

Regression analyses within the T1D group were constrained by low outcome variability; the model produced a pseudo-R^2^ of 0.22, and the precision parameter φ was estimated at 86.1 (95% CI: 56.9–129.8), indicating very tight clustering of engagement scores around the mean. Older age was the only significant factor inversely linked to the engagement score, with each 10-year increase associated with a decrease in engagement (β = −0.15, 95% CI: −0.22–−0.07, *p* < 0.001). Therefore, digital engagement may represent a structurally embedded aspect of disease management rather than behavior driven by fluctuating attitudes or stress-related factors.

### 3.5. Sensitivity Analyses

Linear regression models with untransformed DAUQ scores confirmed the overall trend and pattern of results. In the full T2D model, insulin treatment was the main predictor (β = 0.820, *p* < 0.001), while self-efficacy had an inverse relationship (β = −0.200, *p* = 0.020). TAM was positively linked (β = 0.571, *p* < 0.001), and diabetes-related stress showed a borderline association (β = 0.058, *p* = 0.052). The reduction in stress in the presence of insulin treatment was consistent across different model specifications. In the simplified model (excluding sex, education, and insulin), self-efficacy (β = −0.374, *p* = 0.002), TAM (β = 0.490, *p* = 0.023), and stress (β = 0.402, *p* < 0.001) were significant, although the significance of TAM shifted close to the threshold in one version (*p* = 0.064 for the reduced model), indicating some sensitivity to assumptions about distribution. Adjusted R^2^ was 0.85 for the full model and 0.71 for the simplified one. Quantile regression revealed consistent predictor effects across the engagement range in T2D (Q1–Q3), with no signs of threshold effects. In T1D, all sensitivity analyses similarly failed to identify significant predictors due to limited outcome variance.

## 4. Discussion

The present study demonstrates that factors influencing digital health engagement vary across diabetes types. In T2D, engagement was influenced by a combination of treatment approach, technology acceptance, self-efficacy, and diabetes-related stress. In contrast, engagement in T1D showed less variability and was only weakly related to demographic factors. By considering engagement as a continuous behavioral measure, the results offer a more detailed understanding of how digital technologies are incorporated into everyday diabetes management. 

The finding that insulin treatment was the strongest independent predictor of digital engagement in T2D is clinically coherent. Insulin-treated T2D patients have greater therapeutic complexity (dose adjustments, glucose monitoring requirements) that necessitates more frequent interaction with digital tools. This structural driver substantially attenuated the observed stress–engagement association, suggesting that previous analyses omitting treatment variables may have overestimated the independent role of emotional burden. Future studies examining psychosocial determinants of digital engagement should routinely adjust for treatment characteristics.

Technology acceptance emerged as a significant determinant of engagement levels in T2D. Consistent with technology acceptance principles, individuals who viewed digital health tools as useful and easy to use showed higher engagement [13,14,15]. These results are consistent with earlier studies linking acceptance-related factors to digital health use, but they expand the literature by showing their relevance when engagement is treated as a continuous variable rather than a simple yes/no measure [30,31,32]. Although the strength of the association was reduced in sensitivity analyses using linear regression, the overall trend remained the same. This indicates that attitudes toward technology are important not only for initial adoption but also for determining the level of digital health engagement.

Self-efficacy was significantly associated with digital engagement in T2D, but in an inverse direction. While prior research shows that people with higher self-efficacy tend to engage in more complex self-management behaviors and adhere to behavioral changes over time [17,18,19], these findings suggest a different pattern in the context of digital technologies. Higher perceived self-efficacy was associated with lower levels of digital engagement, possibly reflecting less reliance on digital tools among those who feel confident managing their condition independently. In practice, a one-unit increase in self-efficacy on the 5-point scale predicted a decrease of about 0.25 points on the DAUQ scale. This indicates that digital engagement in T2D might partly serve as a coping strategy for those with lower perceived self-management confidence. Still, other explanations—such as confounding by disease severity, health literacy, or socioeconomic factors—cannot be ruled out given the cross-sectional nature of the study.

In T2D, clinical features such as age and diabetes duration were not independently associated with engagement after adjusting for psychosocial factors. Although age is often considered a key predictor of technology use, the current findings are consistent with growing evidence that chronological age alone is a poor measure of digital engagement when behavioral and attitudinal factors are considered [15,16,20]. Similarly, longer disease duration did not independently forecast engagement, indicating that greater experience with diabetes management does not automatically lead to increased reliance on digital solutions [15,20]. Conversely, in T1D, age remained independently associated with engagement, possibly reflecting generational differences in how technology is integrated within a care model that is heavily dependent on technology.

The near-complete separation between diabetes types and engagement levels reflects system-level determinants rather than purely volitional behavioral differences. Romanian reimbursement policies restrict CGM access primarily to T1D, creating a structural foundation for device-dependent, high-intensity digital engagement in T1D that is largely independent of psychosocial modulation. In T2D, where CGM use was essentially absent (0.5%), engagement variability occurred within a restricted range (DAUQ: 1.71–3.29 for 99% of participants), driven by app-based behaviors (glucose logging, medication tracking) rather than device-generated data integration. This contextual asymmetry has important implications: interventions targeting T1D engagement may yield limited returns given the already-high structural baseline, whereas T2D represents the primary population where psychosocial targeting could meaningfully increase engagement.

### 4.1. Limitations of the Study

Several limitations should be considered when interpreting these findings. First, the cross-sectional design precludes causal inference. The direction of the observed associations—particularly between diabetes-related stress and engagement, and between self-efficacy and engagement—remains ambiguous. Stress may motivate engagement, or engaging with digital tools may influence perceived stress. Longitudinal designs with repeated measures are needed to disentangle temporal dynamics.

Second, recruitment from three outpatient clinics in Western Romania limits generalizability to other healthcare systems. Romanian CGM reimbursement policies, which restrict sensor access primarily to T1D patients, constitute a system-level structural determinant of the observed engagement patterns. These findings may not extrapolate to settings with broader CGM access for T2D populations.

Third, all measures relied on self-report, introducing potential response bias, social desirability, and recall limitations. The DAUQ, while demonstrating strong internal consistency (α = 0.89) and expert-validated content, has not been independently validated in external populations. Future studies should assess convergent validity against objective usage logs (e.g., device download data, app analytics).

Fourth, the psychosocial measures exhibited pronounced ceiling and floor effects. In the T2D stratum, self-efficacy had only 2 unique values (3: *n* = 194; 4: *n* = 6) and the TAM composite had only 2 unique values (1.44: *n* = 199; 2.78: *n* = 1). This extreme distributional concentration inflated Variance Inflation Factors to high levels (self-efficacy VIF = 234.8; TAM VIF = 250.7), not because of true multicollinearity between predictors (bivariate Pearson correlations were modest: self-efficacy–TAM r = 0.40), but because of minimal within-predictor variance. As a result, the T2D regression coefficients are influenced by only a few participants who deviate from typical values, so their interpretation should be approached with caution.

Fifth, the DAUQ composite produces only 8 unique observed values (out of 29 theoretically possible), creating significant discreteness that standard beta regression does not explicitly account for. While beta regression generally outperforms linear regression for bounded outcomes, alternative ordinal methods or zero/one-inflated beta models may be more suitable for highly discrete bounded outcomes and warrant consideration in future research.

Among the strengths of this study are the use of beta regression for a bounded continuous outcome, which represents a methodological improvement over binary adoption measures; the integrated psychosocial–clinical analytical framework with clear treatment adjustment; the stratified analysis that uncovers structurally different engagement mechanisms; and the thorough sensitivity analyses across various model specifications.

### 4.2. Future Research Directions

Several avenues for future research arise from these findings. First, prospective longitudinal studies with repeated DAUQ measurements are necessary to determine the temporal relationships between psychosocial changes and engagement patterns. Second, the DAUQ tool should undergo formal external validation in independent populations, including assessments of test–retest reliability and convergent validity against device-based objective usage data. Third, qualitative or mixed-methods research could shed light on the mechanisms behind the inverse relationship between self-efficacy and engagement, exploring whether this indicates genuine compensatory behavior or unmeasured confounding factors. Fourth, cross-national studies comparing factors influencing engagement across healthcare systems with different reimbursement policies would help clarify the relative roles of structural versus psychosocial determinants. Finally, intervention studies that focus on psychosocial readiness—especially technology acceptance—before implementing digital health solutions could test the causal hypotheses suggested by these cross-sectional findings.

## 5. Conclusions

Digital engagement in diabetes care reflects different underlying mechanisms across disease types. In T1D, engagement is structurally embedded in routine care through device-dependent management methods (e.g., CGM, insulin pumps) and shows limited behavioral variation, with age as the main independent factor. In contrast, in T2D—where technology use is mostly discretionary and device access is restricted by reimbursement policies—the engagement level is influenced by treatment features (insulin use being the strongest predictor), psychosocial factors (such as technology acceptance and self-efficacy), and, to a lesser extent, by diabetes-related stress. The reduction in the stress–engagement link after adjusting for insulin treatment emphasizes the importance of comprehensive covariate selection in digital health research.

These findings have direct implications for clinical practice, digital health implementation, and health policy. For clinicians, the results indicate that psychosocial screening for technology readiness should precede the deployment of digital tools, especially in T2D populations. For digital health program designers, the inverse relationship between self-efficacy and engagement supports the development of adaptive interventions that adjust digital support based on individuals’ confidence levels. For policymakers, the predominant role of reimbursement-linked technology access in shaping engagement patterns highlights the importance of evaluating expanded CGM access for T2D populations showing sufficient digital readiness. Future longitudinal and cross-national studies are necessary to confirm these findings and to test targeted implementation strategies tailored to the unique engagement patterns of T1D and T2D populations.

## Figures and Tables

**Table 1:** Baseline Characteristics Stratified by Level of Digital Engagement.

Variable	Low Engagement (*n* = 196)	High Engagement (*n* = 108)	*p*-Value
*A. Demographic Variables*			
Age, years, median (Q1–Q3)	67.00 (62.00–72.00)	40.00 (32.75–51.25)	<0.001
Male sex, *n* (%)	104 (53.1)	43 (39.8)	0.028
Education level, *n* (%)			<0.001
Primary	7 (3.6)	3 (2.8)	
Secondary	65 (33.2)	7 (6.5)	
High school	93 (47.4)	50 (46.3)	
University	31 (15.8)	48 (44.4)	
*B. Clinical Characteristics*			
Type of diabetes, *n* (%)			<0.001
Type 1 diabetes	0 (0.0)	104 (96.3)	
Type 2 diabetes	196 (100.0)	4 (3.7)	
Insulin treatment, *n* (%)	56 (28.6)	108 (100.0)	<0.001
Diabetes duration, years, median (Q1–Q3)	9.00 (3.00–17.00)	17.00 (10.00–24.25)	<0.001
CGM use, *n* (%)	0 (0.0)	101 (93.5)	<0.001
Insulin pump use, *n* (%)	0 (0.0)	35 (32.4)	<0.001
*C. Digital Health Context*			
Telemedicine use, *n* (%)	133 (67.9)	103 (95.4)	<0.001
Smartphone ownership, *n* (%)	195 (99.5)	108 (100.0)	0.467
*D. Psychosocial Variables*			
Self-efficacy (1–5), median (Q1–Q3)	3.00 (3.00–3.00)	5.00 (5.00–5.00)	<0.001
Technology acceptance (TAM)—Perceived usefulness (1–5), median (Q1–Q3)	1.00 (1.00–1.00)	5.00 (5.00–5.00)	<0.001
Technology acceptance (TAM)—Perceived ease of use (1–5), median (Q1–Q3)	1.00 (1.00–1.00)	5.00 (5.00–5.00)	<0.001
Diabetes-related stress (0–10), median (Q1–Q3)	3.00 (3.00–5.00)	6.00 (6.00–6.00)	<0.001
*E. Summary*			
DAUQ engagement score, median (Q1–Q3)	1.71 (1.71–2.39)	3.86 (3.86–4.43)	<0.001

Note: Low digital engagement is defined as a DAUQ score ≤ median (2.71). Continuous variables compared using the Mann–Whitney U test; categorical variables compared using a chi-square test. SD = standard deviation; CGM = continuous glucose monitoring; TAM = Technology Acceptance Model; DAUQ = Digital Adherence and Use Questionnaire.

**Table 2:** Digital Engagement Patterns by diabetes type (*n* = 304).

Variable	Type 1 Diabetes (*n* = 104)	Type 2 Diabetes (*n* = 200)	*p*-Value
Diabetes duration, years, median (Q1–Q3)	17.00 (9.75–25.25)	9.00 (3.00–17.00)	<0.001
Insulin treatment, *n* (%)	104 (100.0)	60 (30.0)	<0.001
CGM use, *n* (%)	100 (96.2)	1 (0.5)	<0.001
Insulin pump use, *n* (%)	35 (33.7)	0 (0.0)	<0.001
Telemedicine use, *n* (%)	101 (97.1)	135 (67.5)	<0.001
Self-efficacy (1–5), median (Q1–Q3)	5.00 (5.00–5.00)	3.00 (3.00–3.00)	<0.001
Technology acceptance (TAM) score (1–5), median (Q1–Q3)	5.00 (5.00–5.00)	1.00 (1.00–1.00)	<0.001
Diabetes-related stress (0–10), median (Q1–Q3)	6.00 (6.00–6.00)	3.00 (3.00–5.00)	<0.001
DAUQ engagement score (1–5), median (Q1–Q3)	3.86 (3.86–4.43)	1.71 (1.71–2.71)	<0.001

Note: Continuous variables compared using the Mann–Whitney U test; categorical variables compared using the χ^2^ test. SD = standard deviation; CGM = continuous glucose monitoring; TAM = Technology Acceptance Model; DAUQ = Digital Adherence and Use Questionnaire.

**Table 3:** Spearman correlations between DAUQ-based engagement score and study variables stratified by diabetes type.

Variable	Type 1 Diabetes (*n* = 104)		Type 2 Diabetes (*n* = 200)	
	r	*p*-Value	r	*p*-Value
*Demographic variables*				
Age (years)	−0.452	<0.001	0.072	0.314
*Clinical characteristics*				
Diabetes duration (years)	−0.103	0.298	0.156	0.027
*Psychosocial variables*				
Self-efficacy	0.379	<0.001	−0.032	0.654
Technology acceptance	0.379	<0.001	0.075	0.291
Diabetes-related stress	0.187	0.058	0.866	<0.001

Note: r = Spearman correlation coefficient. DAUQ engagement score treated as a continuous outcome. All tests are two-tailed.

**Table 4:** Beta regression models predicting DAUQ-based engagement among individuals with T2D.

Variable	β (95% CI)	*p*-Value
*Demographic variables*		
Age (per 10 years)	0.016 (−0.020 to 0.053)	0.378
Male sex	−0.028 (−0.093 to 0.038)	0.406
Education level	−0.030 (−0.073 to 0.013)	0.165
*Clinical variables*		
Diabetes duration (per 5 years)	−0.02 (−0.06 to 0.05)	0.919
Insulin treatment	0.996 (0.859 to 1.134)	<0.001
*Psychosocial variables*		
Self-efficacy (per unit)	−0.366 (−0.608 to −0.124)	0.003
Technology acceptance (TAM composite) (per unit)	0.694 (0.350 to 1.037)	<0.001
Diabetes-related stress (per unit)	0.069 (0.006 to 0.132)	0.033
*Model fit*		
Precision parameter φ	106.5 (70.5–161.0)	

Note: β = regression coefficient on the logit scale; back-transformed effects are reported in the text for clinical interpretability. Models adjusted for all variables shown. TAM = Technology Acceptance Model.

## Data Availability

De-identified participant data and analysis code are available from the corresponding author upon reasonable request and ethics approval.
